# Assessing the Influence of Hyaluronan Dressing on Wound Healing on Split-Thickness Skin Graft Donor Sites Using a Three-Dimensional Scanner

**DOI:** 10.3390/jcm13216433

**Published:** 2024-10-27

**Authors:** Anna Bock, Florian Peters, Marius Heitzer, Philipp Winnand, Kristian Kniha, Marie Sophie Katz, Frank Hölzle, Ali Modabber

**Affiliations:** 1 Department of Oral and Maxillofacial Surgery, University Hospital RWTH Aachen, 52074 Aachen, Germany; 2 Private Clinic for Oral and Maxillofacial Surgery, 80331 Munich, Germany

**Keywords:** hyaluronan, hyaluronic acid, wound healing, three-dimensional scanning

## Abstract

**Objectives:** The topical application of hyaluronic acid after injury may accelerate the wound healing process. We aimed to retrospectively investigate whether the topical application of hyaluronic acid on standardized wounds after split-thickness skin graft removal on the thigh would accelerate wound healing and improve scarring outcomes. Additionally, we aimed to evaluate the usefulness of three-dimensional (3D) scanning to assess scars. **Methods:** The wound healing process of a hyaluronan group (*n* = 20) and a control (*n* = 21) were analyzed and evaluated using 3D scans at 7 and 14 days and 1, 3, and 6 months post-operatively. Scar evaluations by the patients were conducted 6 months post-operatively using the patient and observer scar assessment scale and the Manchester scar scale. Experts evaluated the scars after 6 months using a modified version of both scales. **Results:** On days 7 and 14, significantly larger areas of the wound surface were closed in the hyaluronan group compared to the control group (*p* < 0.05). After 1 month, significantly more crusted areas remained in the control group than in the hyaluronan group (*p* < 0.05). At the 6-month self-assessments, the hyaluronan group evaluated their scars as being significantly better compared to the control group. **Conclusions:** The topical application of hyaluronic acid in combination with polyurethane foam as a wound dressing after split skin removal accelerated the wound healing rate and positively influenced scar appearance after 6 months. Three-dimensional scanning is useful for evaluating and documenting the wound healing process.

## 1. Introduction

Scars result from injuries to the epidermis and the layers beneath the epidermis. In addition to the size and depth of the wound, the inflammatory reaction and tissue mechanics play important roles in the healing process, the maturation of the scar, and its future appearance. The healing of dermal wounds follows a strict sequence to ensure the skin is rebuilt progressively.

Hyaluronic acid is the main component of the extracellular matrix and acts as a connector between the cellular systems. During the growth of the extracellular matrix, migration, differentiation, and cell proliferation take place [1,2,3]. Hyaluronic acid is responsible for the maintenance of homeostasis in the body and the tonus of tissue. It also regulates the exchange of information and intercellular signal transmission. In humans, hyaluronic acid is omnipresent. Half the total amount is in the skin, as it is produced by dermal fibroblasts and epidermal keratinocytes [1,4,5]. Hyaluronic acid plays a key role in all four phases of wound healing during the skin repair process. In addition to activating and moderating the initial inflammatory reaction, hyaluronic acid influences cell proliferation, cell migration, the function of the keratinocytes, and scarring [1,4,5,6,7].

In recent years, several researchers have investigated the influence of hyaluronic acid on wound healing [8,9,10,11,12,13,14,15,16]. For example, Yildrim et al. showed that hyaluronic acid is a secure and effective treatment for burns, while Cankaya et al. demonstrated that hyaluronic acid improved the healing rate of free gingival grafts [12,15]. In a double-blinded randomized control trial, Dereure et al. found that hyaluronic acid resulted in reduced pain and faster wound healing in a group of patients with venous leg ulcers compared with the control group. Nevertheless, the rate of complete healing was found to be similar in both groups at the secondary endpoint [17].

Superficial uninfected wounds that may benefit from hyaluronic acid treatment are the donor sites from split skin grafts. A split skin graft is defined as a free skin transplant consisting of the epidermis and the superior part of the dermis. The average thickness of this graft is 0.2–0.8 mm. Split skin graft thickness is a crucial factor influencing the pigmentation and quality of the graft. The thinner the split skin graft, the better the chance of successful integration and the more discreet the scar will be on the donor site. However, a disadvantage of thinner grafts is the increased secondary shrinking tendency on the recipient site. Grafts of medium thickness rarely tend to shrink, although they may cause hypertrophic scars or keloids on the donor site. Split skin grafts with a thickness of 0.6–0.8 mm provide the best esthetic results at the donor site, but they have an inferior healing rate [18,19,20]. A dermatome can be used to harvest a split skin graft. Preferred regions for donor sites are the ventrolateral proximal thigh and the inside and outside of the upper arm [20]. The requirement for the donor site should be fast healing with complete re-epithelization. However, to date, there is no evidence-based standard treatment for such wounds. The literature only recommends moist wound management and rare dressing changes [21,22]. The treatment of this wound is an important clinical issue, as patients often report more pain and discomfort and a prolonged healing time at the donor site than at the graft recipients’ site [22,23,24,25]. The aim of this retrospective study was therefore to investigate whether the topical application of hyaluronic acid after split-thickness skin graft removal on the thigh of donors (the hyaluronan group) would accelerate wound healing and improve outcomes compared to a control group. Additionally, the usefulness of 3D scanning in assessing the wound healing process and scarring was evaluated.

## 2. Materials and Methods

### 2.1. Study Design

**Figure 1:**
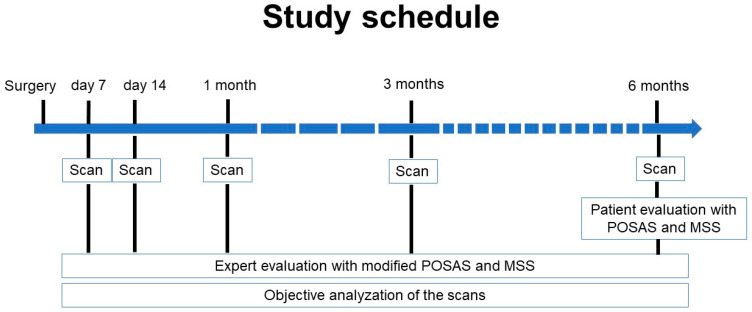
Timing of the study. After 7 and 14 days after surgery, as well as 1, 3, and 6 months after surgery, the wounds are scanned using the Vectra XT 3D imaging system. Six months after surgery, patients evaluate their own wound/scar using the POSAS and MSS. All scans at all time points are evaluated by the experts using the modified POSAS and MSS, and an objective analyzation of all scans took place.

Three-dimensional (3D) images are taken as part of the standard procedure in our clinic for documentation purposes at 7 and 14 days and 1, 3, and 6 months post-operatively. For this study, our database was analyzed retrospectively for patients with microsurgical reconstruction between January 2019 and December 2021. Out of the 274 patients, all the scanning records of the adult patients who underwent standardized skin grafts on the thigh in a single procedure or in combination with other reconstructive surgical procedures were identified (*n* = 116). Patients were excluded if the donor site scans were not taken on day 7 and 6 months post-operatively. Patients were also excluded if more than one scan of the three scans taken between the start and endpoint were missing. Overall, 44 patients were included in this study. The dressing of the wounds changed during the evaluation period as hyaluronic acid was added. Twenty-three patients with a hyaluronan dressing and 21 patients without a hyaluronan dressing were identified from the database. The baseline demographic and clinical characteristics of the patients were collected from their electronic medical records. Furthermore, we analyzed the examiner’s and patients’ evaluations of their scars 6 months post-operatively using the patient and observer scar assessment scale (POSAS) and the Manchester scar scale (MSS). Two experts, each with more than five years of professional experience in oral and maxillofacial surgery, assessed the 3D scans taken during the study period in a blinded manner. They used modified versions of both scales to evaluate the scans on days 7 and 14, as well as 1, 3, and 6 months post-operatively (see Figure 1 and Supplementary Materials). The study protocol was approved by the University Ethics Committee (EK 053/21) and was performed in accordance with the ethical principles of the Declaration of Helsinki.

### 2.2. Wounds and Wound Dressing

The donor site for each split skin graft was located on the patient’s anterolateral thigh. The skin grafts were harvested with a dermatome (AESCULAP^®^, Tuttlingen, Germany) set to a depth of 0.4 mm. Immediately after harvesting, the donor site was covered with a moist compress (saline solution and adrenaline) until the surgery had been completed. At the end of the operation, all the patients received standard wound dressing.

The standard dressing at our clinic consists of a polyurethane foam dressing (Mepilex^R^ XT Foam Dressing, Molnlycke Health Care GmbH, Duesseldorf, Germany) and an adhesive bandage cover (Fixomull, BSN Medical GmbH, Hamburg, Germany). The foam dressing is designed for moderate exuding wounds and has a special adhesive wound contact layer to protect new tissue and intact skin [26,27,28]. The dressing seals the wound margins to protect the skin from damaging leakage and maceration [29]. The standard dressing changes took place on days 7 and 14 and if necessary, on day 21 post-operatively.

As the standard dressing was modified, half of the patients additionally received a hyaluronan application to the standard dressing on days 1, 4, and 7 post-operatively. A combination of hyaluronic acid (50 kDa), perfluorodecalin, and physalis angulata extract (Ready Medical Post-Treatment, SpaMedItaly/Jet Tech Europe S.r.l., Mailand, Italy) was used. For each wound dressing, one pack containing 1.5 mL of the mixture was used. The additional substances that were integrated with the mixture were mainly for viscosity control or as a basis for the main ingredients and comprised aqua, ammonium acryldemethyltaurate/Vp copolymer, alkyl benzoate, cyclopentasiloxane, cyclohexasiloxane, bisabolol, caprylic/Capric, triglyceride, and tocopherol.

### 2.3. Three-Dimensional Imaging

The 3D scanning was performed using the Vectra XT 3D imaging system (Canfield Imaging Systems, Fairfield, NJ, USA), which is used for standard care in our hospital. The camera system is adjustable to body height. It contains six color cameras positioned in a triangulated configuration, which enables 180° images to be captured. The integrated software subsequently processes the images into a high-resolution 3D image model. We used the VECTRA analysis module software (Version 2.0) to process the images. For the detailed analysis of the skin images, the colors red and brown were filtered, which corresponded to the distribution of hemoglobin and melanin in the superficial skin layer [30].

### 2.4. Evaluation Scales

The Manchester scar scale (MSS), proposed by Beausang et al. in 1998, is applicable to a wide range of scars. [31] A visual analog scale (VAS) ranging from excellent to poor is used at the outset for an individual assessment of the scar. The patient assesses and rates the scar parameters, scar color (perfect, slight, obvious, or gross mismatch to surrounding skin), skin texture (matte or shiny), relationship to surrounding skin (range from flush to keloid), texture (range of normal to hard), and distortion (range of none to severe). All the scores are added together to give an overall score for the scar ranging from 5 to 18, with low scores representing clinically better scars [31,32]. Because the experts evaluated the scars using the 3D images, the MSS was modified for them to include only the VAS, scar color, and skin texture, which in turn led to changed scoring that ranged from 2 to 6 (scoring sheet, see Supplementary Digital Material Content 1).

The patient and observer scar assessment scale (POSAS) focuses on scar severity from both the clinicians’ and patients’ point of view [33]. It consists of two numeric scales. The patient scar assessment scale includes the subjective symptoms of pain and pruritus and assesses the patient’s perceptions of the scar color, stiffness, thickness, and homogeneity. The observer scar assessment scale is used to evaluate vascularity, pigmentation, thickness, relief, and pliability. Both scales range from 5 to 50, with high scores representing clinically worse scars [31,33]. In this study, the observer scar assessment scale was modified to include vascularity, pigmentation, and homogeneity, which resulted in a range from 3 to 30 (scoring sheet, see Supplementary Digital Material Content 2).

### 2.5. Wound Healing Process Analysis

For an objective analysis of the wound healing process, the images were evaluated by two experts using the HTML application SCalAr to determine the areas of closed, crusting, and open wounds. After uploading the images, the corresponding areas were marked, and the percentage of the overall wound area could be determined. The mean values determined by the two experts were used for further analysis.

Furthermore, for an objective analysis of the red and brown filtered images, the free software GNU Image Manipulation Program (GIMP 2.10.30) was used. After transforming the images to greyscale mode, the black channel was used, as it is able to show 256 different values, where 0 represents the lowest intensity (black) and 255 the highest (white). After marking the wound area, the data from the histogram (i.e., mean, standard deviation) for the selected areas were determined and compared. The histogram visualized the distribution of the brightness values of the image. The average value of the histogram indicated the percentage of the total tonal range (0–255) in which the majority of the pixels were represented.

### 2.6. Statistical Analysis

The obtained data were arranged using MS Office Excel 2016^®^ (Microsoft Corporation, Redmond, WA, USA). The statistical analyses were conducted using SPSS version 28 (SPSS, IBM, Armonk, NY, USA) and GraphPad Prism 6 Software (GraphPad Software, San Diego, CA, USA). Cohen’s weighted Kappa was used to evaluate the interrater reliability of the experts. Values ≤0 indicated no agreement, 0.01–0.20 indicated none to slight agreement, 0.21–0.40 indicated fair agreement, 0.41–0.60 indicated moderate agreement, 0.61–0.80 indicated substantial agreement, and 0.81–1.00 indicated almost perfect agreement. The normal distribution was checked using the D’Agostino–Pearson normality test in the omnibus K2 variant. Baseline characteristics were given as median values with interquartile ranges or numbers with percentages. Differences in baseline characteristics between the intervention and control group were analyzed using the chi-square test for sex and the ASA score or the Mann–Whitney test for age and BMI. Study variables were described as median values with interquartile ranges (IQRs) and were described separately for the intervention and control group. Differences in study variables between the intervention and control group were analyzed using the Mann–Whitney test. Differences between control and hypothetical values of 1.0 (wound closed) or 0.0 (wound crusted and wound open) were tested using the Wilcoxon signed-rank test. The median Cohen’s Kappa value was 0.471 between the two experts evaluating the VAS, color, surface, vascularization, and pigmentation after 7 days, 14 days, 1 month, 3 months, and 6 months. Statistical significance was assumed at *p* values < 0.05.

## 3. Results

### 3.1. Participants

A total of 41 patients who underwent a skin graft on the thigh in a single procedure or in combination with other reconstructive surgery procedures were included in the study. Out of the 23 participants in the hyaluronan group, 13 were male and 10 were female. Their median age was 60 years (IQR = 15), and the mean wound size was 28.2 cm^2^ (SD = 11.76). In the control group (*n* = 21) were 11 male and 10 female participants. The median age of this group was 59 years (IQR = 14) and the mean wound size was 25.69 cm^2^ (SD = 10.53 cm^2^). These results and other baseline characteristics, such as body mass index and the ASA score are shown in Table 1.

### 3.2. Wound Healing Process

**Figure 2:**
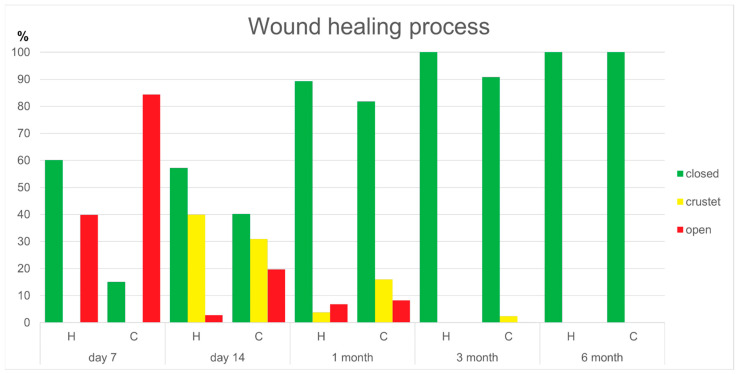
The hyaluronan group (H) and the control group (C) showed significant differences in the healing process.

The healing stages showed significant differences between the groups. On day 7, significantly larger areas of the wound surface were closed in the hyaluronan group compared to the control group (*p* = 0.003). On day 14, there were significantly more areas still open and exuding in the control group than in the hyaluronan group (*p* = 0.024). After 1 month, there were significantly more crusted and open areas in the control group than in the hyaluronan group (*p* = 0.032 and *p* = 0.016). However, after 3 and 6 months, there were no significant differences in the healing process between the groups. The results of the wound healing process are shown in Figure 2 and Table 2.

**Figure 3:**
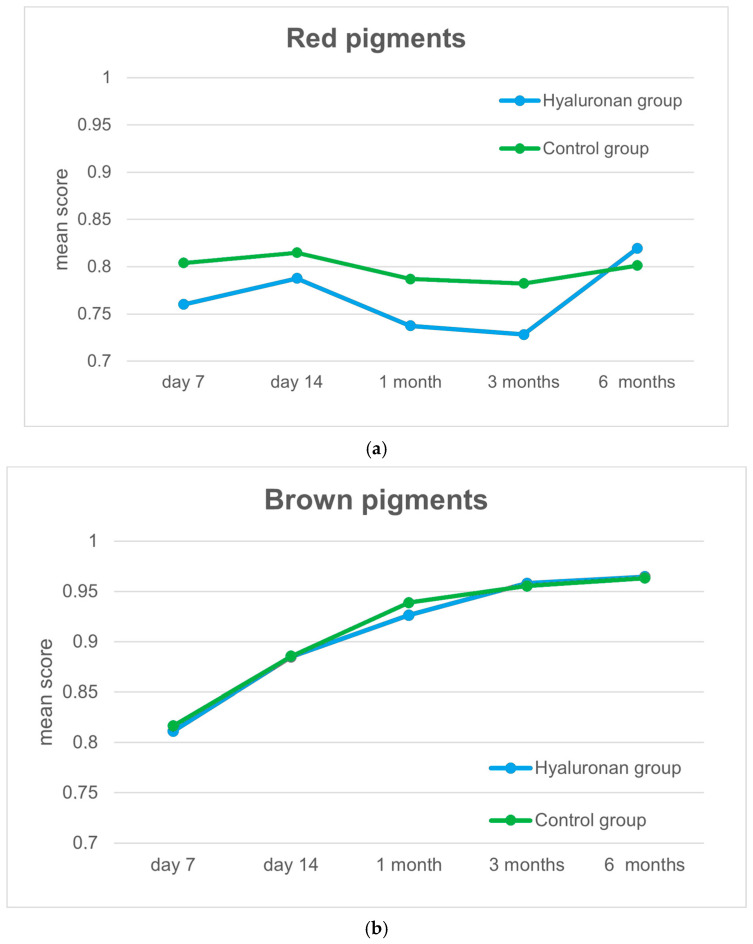
(**a**) The hyaluronan group started with a lower score (i.e., a darker gray tone and therefore a higher density of red pigments in the wound area). After 6 months, the hyaluronan group had a lower density of red pigments (i.e., a lighter shade of gray than the control group). (**b**) Both groups showed an equal distribution of brown pigments in the wound area throughout the healing process.

In terms of the red and brown filtered images during the healing process, significant differences in the distribution of the red pigments were evident on day 7 and at 1 and 3 months. No significant differences were apparent in the distribution of the brown pigments at any time during the healing process. The results are shown in Figure 3.

**Figure 4:**
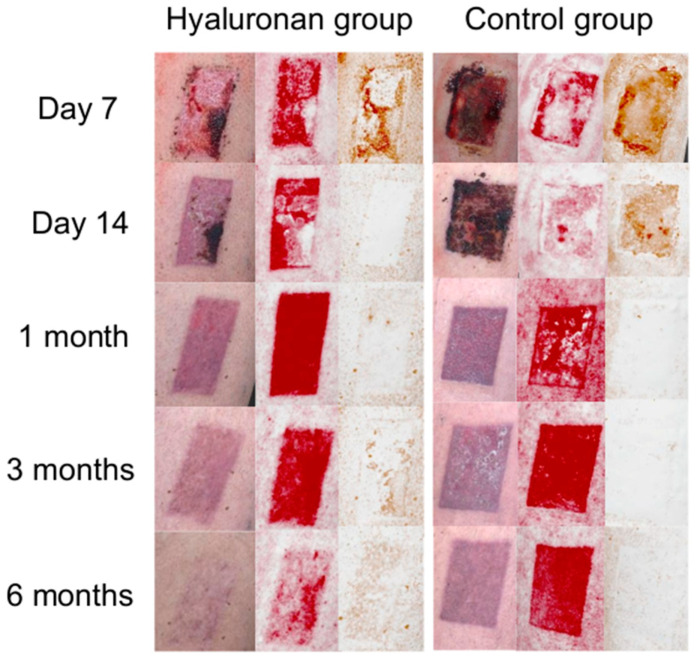
Comparison of the wound healing processes for the hyaluronan and control groups using clinical examples. On the left, the 3D images of the wounds are shown. In the middle, the red filtered images are shown and on the right, the brown filtered images are shown.

The experts evaluated all the scar images (Figure 4) in terms of the VAS and color and assessed whether they were matte vs. shiny. Additionally, they evaluated the vascularization and pigmentation. All the results are shown in Table 3.

The experts also evaluated the homogeneity of the scars after 6 months. The mean score for the hyaluronan group was 2.89 (SD = 1.74) and 3.8 (SD = 1.66) for the control group. There was no significant difference between the groups.

The median Cohen’s Kappa in this study was considered as demonstrating moderate agreement.

### 3.3. Patients’ Scar Outcome Assessments After 6 Months

**Figure 5:**
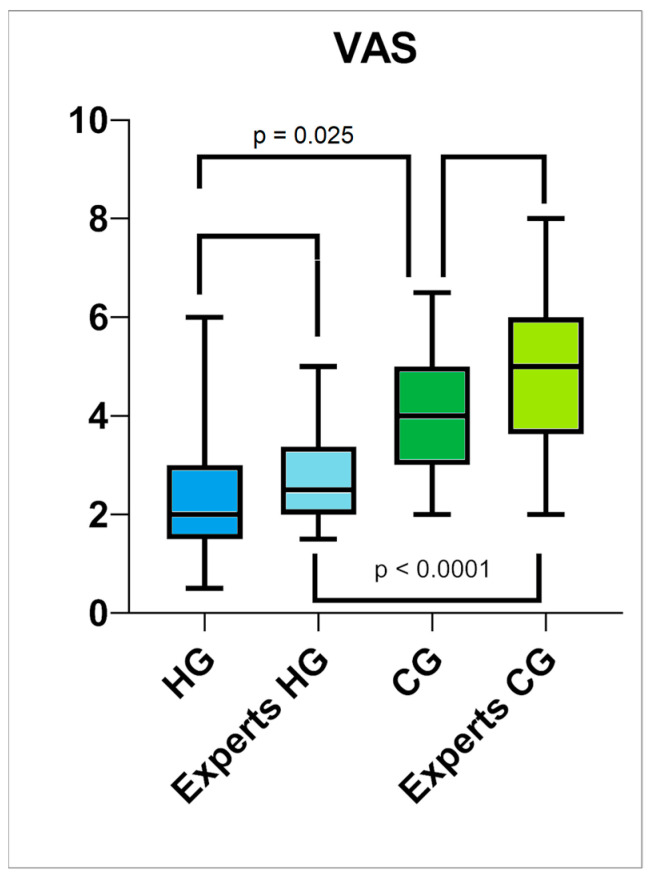
Box plot comparing the results of the overall outcomes (VAS) of the scars according to the patients and experts after 6 months and the period in between. Differences between the intervention and control were analyzed using the Mann–Whitney test. Like the experts, the patients in the hyaluronan group rated the overall outcomes of their scars as being significantly better compared to the control group. There were no significant differences between the assessments of the patients in the hyaluronan and control groups and those of the experts.

The hyaluronan group evaluated their scars as significantly better compared to the control group during the self-assessment after 6 months. The mean overall score for the MSS in the hyaluronan group was 6.7 (SD = 0.35) and 8.67 (SD = 0.83) in the control group (*p* < 0.0001), while that for the POSAS was 12.83 (SD = 1.25) for the hyaluronan group and 16.81 (SD = 1.73) for the control group (*p* = 0.0134). More specifically, the groups showed significant differences in the categories of the overall appearance of the scar (VAS) (see Figure 5), color, matte vs. shiny, and homogeneity. All the results are shown in Table 4.

### 3.4. Examiner’s Scar Outcome Assessment after 6 Months

The examiners evaluated the scars of the hyaluronan group as significantly better compared to the control group during the self-assessment after 6 months. The mean overall score for the MSS for the hyaluronan group was nine (SD = 0.76) and sixteen (SD = 2.11) in the control group (*p* > 0.0001), while that for the POSAS was eight (SD = 0.8) for the hyaluronan group and sixteen (SD = 2.86) for the control group (*p* < 0.0001).

More specifically, the groups showed significant differences in the categories of the overall appearance of the scar (VAS), color, surface, vascularization, and pigmentation. All the results are shown in Table 5.

### 3.5. Comparison of the Patients’ and Experts’ Evaluations 6 Months Post-Operatively

There were no significant differences between the hyaluronan group and the experts with respect to the VAS (*p* = 0.0646), color (*p* = 0.2496), matte vs. shiny (*p* = 0.1716), and homogeneity (*p* = 0.1599) at 6 months. The experts and the control group’s evaluations were not significantly different in terms of the aspects of matte vs. shiny (*p* = 0.1312) and homogeneity (*p* = 0.2912). However, color was rated significantly better by the experts than by the control group (*p* = 0.0002).

## 4. Discussion

The type of wound dressing plays a crucial role in the healing process after split skin graft removal. Several studies have found moist wound healing dressing products to be superior to non-moist products in terms of healing. While numerous dressing types are available, no superior dressing type has been identified to date [22]. Furthermore, the combination of moist wound dressings and additional applications has not yet been investigated. Hyaluronic acid plays a key role in all phases of wound healing. The initial inflammatory reaction is activated and moderated by the hyaluronan as it forms the architectural matrix for the deposition of clotted fibrin. Because of its negative charge, hyaluronan is accompanied by an enormous water domain, which causes an expansion of tissue (i.e., the ‘tumor’ of the inflammatory response). The created space facilitates the migration of other inflammatory cells. Furthermore, hyaluronan induces expressions of chemokines involved in the healing process (i.e., for angiogenesis) and functions as a mediator of the crosstalk between the wound extracellular matrix and the incoming inflammatory cells. In the final stages of the healing process, hyaluronan influences the function and migration of fibroblasts and stimulates collagen production [34]. Recently, several studies have shown a positive effect with the topical application of hyaluronic acid on different wound healing sites (i.e., burns or chronic ulcers) [8,9,10,11,12,13,14,15,16]. In this study, we intended to investigate whether the application of hyaluronic acid influences the wound healing process and to evaluate the outcome of scars after split skin removal on the thigh after 6 months. In addition, 3D scanning was evaluated for its usefulness in assessing the wound healing process and scars.

### 4.1. Wound Healing Process

The objective analysis of the 3D images during the study period showed that the wounds of the hyaluronan group healed significantly faster than those of the control group. On day 7, an average of 60.11% of the wounds in the patients who received hyaluronic acid application had closed but only 15.06% had closed in the control group. On day 14, 97.16% of the hyaluronan group’s wounds had closed/crusted versus only 71.13% in the control group. In a study by Kazanavičius et al., different wound dressings on split-thickness skin graft donor sites were compared. In the group that received a dressing of polyurethane foam (as in this study), 25% of the wounds had healed after 9 days, which is similar to the results of this study [35].

In a study by Francesco et al., the treatment of different wounds with hyaluronic acid was investigated. They found that the re-epithelization of the wounds was accelerated, just as it was confirmed in our study. In contrast to our study, however, different wounds (superficial trauma wounds, surgical sutures, first- and second-degree burns, dermabrasions, ulcers) were compared here, whereas only standardized wounds with the same depth (0.4 mm) were used in our case [36].

Depending on its molecular weight, hyaluronan possesses diverse effects. Especially low-molecular-weight hyaluronan (10–250 kDa), which activates inflammatory and immune responses and shows a significantly higher skin penetration than high-molecular-weight hyaluronan [37]. In this study, the hyaluronan molecular weight was 50 kDa and therefore, a high penetration into the wound can be assumed. As the exact recipe of the product (Ready Medical Post-Treatment) is not published, the effects of the single ingredients and their interactions remain unclear.

The objectively measured accelerated wound healing process of the hyaluronan group and therefore the appearance of the wound/scar was in agreement with the assessments of the experts during the study period. All images of the hyaluronan group were rated as significantly better than those of the control group with respect to visual appearance and color compared to the surrounding skin. After 3 and 6 months, the hyaluronan group’s scars also appeared significantly more matte. As the basic conditions were the same for both groups, it can be assumed that the accelerated wound healing rate was due to the topical application of hyaluronic acid. This would correspond with the results of previous studies, which have shown that hyaluronic acid and perfluorodecalin can significantly improve the epithelization rate of wounds [15,38].

Due to the study design, it was not possible to determine the precise date of complete healing. Previous studies had equal circumstances that resulted in the approximation of days to heal [22,39,40]. For example, in a study by Kazanavičius et al., the mean healing time after split skin graft removal with moist wound dressings was 9–12 days [35]. In a study by Healy et al., the mean time to epithelialization was 35 days [41]. Notably, wound epithelialization was defined and assessed inconsistently across the studies [42]. We therefore refrained from providing potentially inaccurate information about the complete healing time in this study.

For a further objective assessment, the 3D images were filtered for the colors red and brown, which are supposed to display the distribution of hemoglobin and melanin. It must be kept in mind that the images for days 7 and 14 could only be compared to the other images during the study period to a limited extent, as at this point, exuding or crusted areas would have remained, which would have influenced the results. The brown filtered images did not show significant differences between both groups during the study period. The red filtered images scored significantly lower after 1 and 3 months in the hyaluronan group, which corresponds to higher hemoglobin concentrations. After 6 months, the ratios were reversed, and the hyaluronan group had a paler appearance than the control group. As the local concentrations of hemoglobin in scars correspond to local blood circulation, it can be concluded that the patients who received the hyaluronic acid treatment after split skin graft removal initially had increased vascularization of the scar. The increased blood circulation led to an accelerated cell migration and interaction. This in turn accelerated the tissue remodeling process, which resulted in a paler scar after 6 months [1,7,43].

There are several different types of scarring that are known, i.e., hypertrophic, keloid, or atrophic scars. Hypertrophic and keloid scars occur from a moderate to excessive overproduction of collagen during healing [43]. In this study, none of the participants had risk factors for specific scar development and all scars were ultimately normothropic. In future studies, it would be interesting to investigate the influence of topical hyaluronan applications on patients with risk factors for keloid or atrophic scar developments.

### 4.2. Scar Outcomes

Based on the overall score of the MSS and POSAS, the participants of the hyaluronan group rated their scars after 6 months as significantly better compared to the control group. In addition to the better overall appearance (VAS), the scars were more matte and the color was more like normal skin than in the control group. Apart from that, the hyaluronan group rated the appearance of their scars as more homogeneous than the control group. These assessments were in agreement with the experts’ evaluations of the scars after 6 months.

The patients also assessed the categories of contour, distortion, and texture in the MSS and stiffness and thickness in the POSAS. As expected, these categories were rated equally well in both groups. This is due to the nature of the scars, which were very superficial and flat. If there is no infection to the wound, then no keloid, distortion, or altered textures are expected in such scars. The categories of pain and itchiness were also evaluated as equally good in both groups after 6 months. It would be interesting to investigate in future studies whether these two aspects could be evaluated as equally good at the beginning of the wound healing process, as they play a more important role at that time.

### 4.3. Experts’ Assessments

The analysis of the interrater reliability showed substantial to perfect agreement for the aspects of the VAS, color, and matte vs. shiny. On day 7, only slight to fair agreement between the experts was possible for the aspects of vascularization and pigmentation. This could be explained by the early state of wound healing, which is characterized by exuding and crusted areas. At this point, wounds are very inhomogeneous, which makes assessments difficult. The aspect of pigmentation also showed a lower agreement between the experts than the other aspects during the study period. In this study, all the participants were white, and even normal skin showed just light pigmentation. As the contrasts between the normal skin and the scars were minimal over the course of the study, the assessments were more difficult and led to lower agreement between the experts.

### 4.4. Three-Dimensional Scanner

Three-dimensional scanners have been described as reliable devices for providing documentation during daily medical routines [44]. In addition to providing documentation, a 3D scanner was used to analyze the wounds and scars in this study. The experts evaluated the wounds and scars using only 3D scans and demonstrated high interrater reliability for most aspects. A comparison of the participants’ ‘real’ evaluations of their scars after 6 months and the experts’ evaluations using 3D scans showed similar results without significant differences. Nevertheless, it must be emphasized that the aspects of contour, stiffness, and distortion play an important role in the evaluation of scars and these cannot be evaluated with a 3D scan.

### 4.5. Limitations

In the present study, an aspect that may be considered controversial is the time and frequency of the hyaluronic acid application. Compared to the control group, the group receiving hyaluronic acid received two additional early dressing changes (i.e., on the first and fourth days post-operatively). Generally, dressing changes after split skin removal should be infrequent to support re-epithelization [21,22]. The rate of dressing changes in the present study therefore contradicted contemporary recommendations. The chosen patient collective all underwent microvascular surgery and accordingly received intra- and post-operative anticoagulative therapy. Due to bleeding, an immediate application of hyaluronic acid after split skin removal was not possible and would have been inefficient. Day 1 after the operation was thus chosen for the first application of hyaluronic acid. To accelerate the effect of hyaluronic acid, day 4 after surgery was selected for the second application, because a dressing change would have been provided as a standard procedure for both groups on day 7 after surgery. Future research with a suitable patient cohort should investigate whether a single application of hyaluronic acid immediately after split skin removal would provide similar effects to those in the present study.

In terms of the costs, the hyaluronic acid product used in this study was expensive, at EUR 50 for 1.5 mL of the serum. Given that three applications were required per wound, the dressing of the donor site was EUR 150 more expensive for the hyaluronan group than the control group. Nevertheless, it must be taken into account that thanks to the faster healing rate, the hyaluronan group received fewer dressing changes and costs for the number of consultations and dressing materials were therefore reduced [22,45]. A possible way to reduce the costs further could be to use a single application of hyaluronic acid. A future study could thus investigate whether a single application of hyaluronic acid would provide a similar effect to three applications. Alternatively, a different hyaluronic acid product could be used.

Another limitation of the present study was that the final assessment of the scars took place after 6 months, even though remodeling and the completion of scar healing can take up to 2 years [46]. Other studies have chosen the same follow-up times, as it is difficult to carry out a longer observation period in this group of patients [12]. Nevertheless, it would be interesting to investigate whether the outcomes after 2 years would be the same for both groups.

As far it is known, there are differences in wound healing in white skin and colored skin. Individuals with colored skin have a predisposition for keloid formation and altered pigmentation [47]. In this study, only individuals with white skin have been included due to the ability to evaluate the 3D scans. Studies including individuals with all skin color types are necessary in the future.

## 5. Conclusions

The present study suggests that the topical application of hyaluronic acid in combination with polyurethane foam as a wound dressing after split skin removal accelerates the wound healing rate and positively influences scar appearance after 6 months. In the future, randomized controlled studies would be useful to investigate the effect more precisely.

## Figures and Tables

**Table 1:** Baseline characteristics of the participants. Values are described as median values with interquartile ranges. Differences between intervention and control group in age and BMI were tested using the Mann–Whitney test. Differences between intervention and control in sex and ASA were tested using the Chi squared test.

Variable	Intervention	Control	*p* Value
Age (years)	60.0 (15)	59.0 (14)	0.689
Body mass index (kg/m^2^)	24.7 (7.6)	27.5 (5.9)	0.133
Sex (*n*)			
Male	13	11	0.783
Female	10	10	
ASA (*n*)			
≤2	15	11	0.387
>2	8	10	

**Table 2:** Wound characteristics after 7 days, 14 days, 1 month, 3 months, and 6 months. Parameters are indicated as median values (with interquartile ranges). Differences between intervention and control after 7 days, 14 days, and 1 month were analyzed using the Mann–Whitney test. Differences between control and hypothetical values of 1.0 (wound closed) or 0.0 (wound crusted and wound open) were tested using the Wilcoxon signed-rank test. After 6 months, values were identical between control and intervention, so *p* values were not available (n.a.). Significant *p* values are in bold.

Variable	Intervention	Control	*p* Value
AW7D wound closed	0.597 (0.5)	0.128 (0.4)	0.003
AW14D wound closed	0.603 (0.3)	0.377 (0.5)	0.181
AW14D wound crusted	0.364 (0.3)	0.297 (0.6)	0.545
AW14D wound open	0.000 (0.0)	0.037 (0.3)	**0.024**
AW1M wound closed	1.000 (0.2)	0.814 (0.3)	**0.015**
AW1M wound crusted	0.000 (0.0)	0.105 (0.2)	**0.032**
AW1M wound open	0.000 (0.0)	0.015 (0.2)	**0.016**
AW3M wound closed	1.000 (0.0)	1.000 (0.0)	0.250
AW3M wound crusted	0.000 (0.0)	0.000 (0.0)	0.250
AW3M wound open	0.000 (0.0)	0.000 (0.0)	1.000
AW6M wound closed	1.000 (0.0)	1.000 (0.0)	n.a.
AW6M wound crusted	0.000 (0.0)	0.000 (0.0)	n.a.
AW6M wound open	0.000 (0.0)	0.000 (0.0)	n.a.

**Table 3:** Evaluation of the scar images at all time points by the experts. Parameters were indicated as median values (with interquartile ranges). Differences between intervention and control were analyzed using the Mann–Whitney test. Significant *p* values are bold. The visual analog scale (VAS) ranging from excellent to poor (1–10), scar color ranging from perfect, slight, obvious to gross mismatch to surrounding skin (1–4), the skin texture ranging from matte or shiny (1–2), vascularization and pigmentation ranging from normal skin to very different (1–10) were used at the outset for an individual assessment of the scar.

Variable	Intervention	Control	*p* Value
AW7 VAS	8.0 (1.0)	10.0 (1.0)	<0.001
AW7 Color	4.0 (0.5)	4.0 (0.0)	0.022
AW7 Surface	2.0 (0.0)	2.0 (0.0)	0.792
AW7 Vascularization	7.5 (1.0)	8.0 (0.9)	0.002
AW7 Pigmentation	8.0 (0.0)	8.5 (1.0)	<0.001
AW14 VAS	7.0 (2.0)	8.0 (1.5)	<0.001
AW14 Color	3.5 (0.0)	3.5 (0.5)	0.040
AW14 Surface	1.0 (1.0)	1.0 (1.0)	0.837
AW14 Vascularization	7.0 (1.5)	7.5 (1.0)	0.040
AW14 Pigmentation	7.0 (1.0)	7.5 (1.5)	0.391
AW1M VAS	6.75 (1.4)	7.75 (1.5)	0.001
AW1M Color	3.5 (0.5)	3.5 (0.0)	0.382
AW1M Surface	1.0 (0.5)	1.5 (0.5)	0.445
AW1M Vascularization	7.5 (0.9)	7.5 (1.4)	0.724
AW1M Pigmentation	5.25 (1.4)	6.0 (0.5)	0.007
AW3M VAS	5.0 (1.5)	6.75 (1.9)	<0.001
AW3M Color	2.5 (0.0)	3.5 (0.9)	0.006
AW3M Surface	1.0 (0.0)	1.5 (0.5)	0.002
AW3M Vascularization	6.0 (1.0)	6.5 (2.0)	0.423
AW3M Pigmentation	4.0 (0.5)	5.5 (0.9)	<0.001
AW6M VAS	2.5 (1.5)	5.0 (2.5)	<0.001
AW6M Color	1.5 (1.0)	2.5 (0.5)	0.002
AW6M Surface	1.0 (0.0)	1.5 (0.5)	<0.001
AW6M Vascularization	3.5 (2.0)	5.0 (3.5)	0.037
AW6M Pigmentation	3.0 (2.0)	4.0 (1.0)	0.103

**Table 4:** Evaluation of the scar by means of the MSS and POSAS by the participants of the hyaluronan and control group after 6 months. Parameters are indicated as median values (with interquartile ranges). Differences between intervention and control were analyzed using the Mann–Whitney test. Significant *p* values are bold. A visual analog scale (VAS) ranging from excellent to poor is used at the outset for an individual assessment of the scar (1–10). The patient assesses and rates the scar parameters, scar color (perfect, slight, obvious, or gross mismatch to surrounding skin, 1–4), skin texture (matte or shiny, 1–2), relationship to surrounding skin (range from flush to keloid, 1–4), texture (range of normal to hard, 1–4), and distortion (range of none to severe, 1–4). The other aspects, pain and pruritus, scar color, stiffness, thickness, and homogeneity, are rated on a scale from 1 to 10 (1 = no, not at all/just like normal skin, 10 = yes, very much/very different).

Variable	Intervention	Control	*p* Value
MSS VAS	2.0 (1.5)	4.0 (2.5)	<0.001
MSS Color	2.0 (2.0)	3.0 (1.0)	<0.001
MSS Surface	1.0 (0.0)	2.0 (1.0)	0.003
MSS Contour	1.0 (0.0)	1.0 (0.0)	0.591
MSS Distortion	1.0 (0.0)	1.0 (0.0)	0.345
MSS Texture	1.0 (0.0)	1.0 (1.0)	0.121
POSAS Painful	1.0 (0.0)	1.0 (0.0)	0.974
POSAS Itching	1.0 (0.0)	1.0 (0.0)	0.334
POSAS Color different	4.0 (6.0)	5.0 (5.5)	0.293
POSAS Stiffness	1.0 (1.0)	2.0 (2.5)	0.304
POSAS Thickness	1.0 (1.0)	1.0 (1.0)	0.482
POSAS Irregular	1.0 (1.0)	4.0 (5.5)	0.002

**Table 5:** Examiner’s assessment of the scar after 6 months. Parameters are indicated as median values (with interquartile ranges). Differences between intervention and control were analyzed using the Mann–Whitney test. A visual analog scale (VAS) ranging from excellent to poor is used at the outset for an individual assessment of the scar (1–10). The patient assesses and rates the scar parameters, scar color (perfect, slight, obvious, or gross mismatch to surrounding skin, 1–4), skin texture (matte or shiny, 1–2), relationship to surrounding skin (range from flush to keloid, 1–4), texture (range of normal to hard, 1–4), and distortion (range of none to severe, 1–4). The other aspects, vascularization, pigmentation, thickness, relief, and pliability, are rated on a scale from 1 to 10 (1 = no, not at all/just like normal skin, 10 = yes, very much/very different).

Variable	Intervention	Control	*p* Value
MSS VAS	3.0 (2.0)	7.0 (4.0)	<0.001
MSS Color	2.0 (1.0)	4.0 (1.0)	<0.001
MSS Surface	1.0 (0.0)	2.0 (0.5)	<0.001
MSS Contour	1.0 (0.0)	1.0 (0.0)	0.323
MSS Distortion	1.0 (0.0)	1.0 (0.0)	0.888
MSS Texture	1.0 (1.0)	1.0 (1.0)	0.941
POSAS Vascularization	2.0 (4.0)	8.0 (4.5)	<0.001
POSAS Pigmentation	3.0 (2.0)	5.0 (4.0)	<0.001
POSAS Thickness	1.0 (0.0)	1.0 (0.0)	0.753
POSAS Relief	1.0 (1.0)	1.0 (0.5)	0.410
POSAS Pliability	1.0 (1.0)	1.0 (0.0)	0.577

## Data Availability

The data that support the findings of this study are available from the corresponding author, [A.B.], upon reasonable request.

## Supplementary Materials

The following supporting information can be downloaded at: https://www.mdpi.com/article/10.3390/jcm13216433/s1. The experts’ and patients’ assessment forms are supplied as supplementary materials.
