# Supplementary material for: Assessing the Influence of Hyaluronan Dressing on Wound Healing on Split-Thickness Skin Graft Donor Sites Using a Three-Dimensional Scanner
Source: J Clin Med. 2024 Oct 27;13(21):6433. doi: 10.3390/jcm13216433 (PMC11546088; doi:10.3390/jcm13216433)
Supplement: Supplementary file 1 [file jcm-13-06433-s001.zip › Content 2.pdf]

Assessment Expert

Date:

Name:

<u>Modified MSS</u>

Visual Analog Scale

Excellent ⟷ Poor

Color	Perfect	1
	Slight mismatch	2
	Obvious mismatch	3
	Gross mismatch	4
Matt vs. shiny	Matt	1
	Shiny	2

<u>Modified POSAS</u>

	Normal skin									Worst scar imaginable
	1	2	3	4	5	6	7	8	9	10
Vascularization										
Pigmentation										
